# Early Trauma and Increased Risk for Physical Aggression during Adulthood: The Moderating Role of MAOA Genotype

**DOI:** 10.1371/journal.pone.0000486

**Published:** 2007-05-30

**Authors:** Giovanni Frazzetto, Giorgio Di Lorenzo, Valeria Carola, Luca Proietti, Ewa Sokolowska, Alberto Siracusano, Cornelius Gross, Alfonso Troisi

**Affiliations:** 1 European Molecular Biology Laboratory (EMBL), Mouse Biology Unit, Monterotondo, Italy; 2 Department of Neurosciences, University of Rome Tor Vergata, Rome, Italy; James Cook University, Australia

## Abstract

Previous research has reported that a functional polymorphism in the monoamine oxidase A (MAOA) gene promoter can moderate the association between early life adversity and increased risk for violence and antisocial behavior. In this study of a combined population of psychiatric outpatients and healthy volunteers (N = 235), we tested the hypothesis that MAOA genotype moderates the association between early traumatic life events (ETLE) experienced during the first 15 years of life and the display of physical aggression during adulthood, as assessed by the Aggression Questionnaire. An ANOVA model including gender, exposure to early trauma, and MAOA genotype as between-subjects factors showed significant MAOA×ETLE (F_1,227_ = 8.20, P = 0.005) and gender×MAOA×ETLE (F_1,227_ = 7.04, P = 0.009) interaction effects. Physical aggression scores were higher in men who had experienced early traumatic life events and who carried the low MAOA activity allele (MAOA-L). We repeated the analysis in the subgroup of healthy volunteers (N = 145) to exclude that the observed G×E interactions were due to the inclusion of psychiatric patients in our sample and were not generalizable to the population at large. The results for the subgroup of healthy volunteers were identical to those for the entire sample. The cumulative variance in the physical aggression score explained by the ANOVA effects involving the MAOA polymorphism was 6.6% in the entire sample and 12.1% in the sub-sample of healthy volunteers. Our results support the hypothesis that, when combined with exposure to early traumatic life events, low MAOA activity is a significant risk factor for aggressive behavior during adulthood and suggest that the use of dimensional measures focusing on behavioral aspects of aggression may increase the likelihood of detecting significant gene-by-environment interactions in studies of MAOA-related aggression.

## Introduction

Expressions of violent behavior such as aggression are influenced by a complex and dynamic interplay of biological, psychological and social variables. Individual differences in aggressive behavior are at least partly heritable and presumably result from the interaction between genetic and environmental factors [1]. Gene-environment interactions (G×E) refer to genetic differences in susceptibility to particular environmental risk factors. It is well documented that early life environmental risk factors have detrimental effects on the long-term mental health of individuals and increasing evidence suggests that genotype can moderate the capacity of early environmental pathogens to alter risk for mental disorders [2]. In the development of adult antisocial and violent behavior, the environmental factors considered influential include *in utero* exposure to pathogens and birth complications [3], childhood abuse or neglect [4], [5], and family relationships, home environment, and other social variables [6].

The clearest genetic link to aggressive behavior exists for the monoamine oxidase A (MAOA) gene which plays a key role in the catabolism of monoamines, including dopamine (DA), norepinephrine (NE), and serotonin (5-HT) [7]. MAOA knockout (KO) mice display elevated levels of DA, NE and 5-HT and male KO mice exhibit increased aggressive behavior [8]. Forebrain-restricted transgenic expression of MAOA in MAOA KO mice results in lower levels of DA, NE and 5-HT and in a reversal of the aggressive phenotype, suggesting that lack of enzyme activity in the forebrain of MAOA KO mice underlies their behavioral phenotype [9]. In humans, a missense mutation was found in the MAOA gene (Xp11.23-11.4) in a Dutch kindred whose members exhibited a pattern of impulsively violent behavior for generations [10]. Since MAOA is an X-linked gene [11], hemizygous males from this family effectively represent functional gene knockouts. However, this mutation is extremely rare and has not been found in any other pedigree. More recently, a common variable number tandem repeat (VNTR) polymorphism lying 1.2 kb upstream of the transcription initiation site of MAOA has been shown to affect transcriptional activity of the gene in transfected cells. The 3.5-and 4-repeat forms show high MAOA mRNA expression and high enzyme activity, while the 2-, 3-and 5-repeat forms show low MAOA mRNA expression and low enzyme activity [12], [13].

Recently, a large longitudinal study of children followed for 26 years since birth showed that MAOA VNTR genotype moderates the association between childhood maltreatment and violent and antisocial behavior [14]. Although several studies have replicated this G×E effect [15]–[17], other studies have failed to replicate the original findings reported by Caspi and co-workers [18]–[20]. Explanations for these conflicting findings are manifold, including the use of different measures of the behavioral phenotype and of environmental risk factors. In this work, we studied a mixed population of psychiatric outpatients and healthy volunteers to test the hypothesis that the MAOA VNTR polymorphism moderates the association between early traumatic life events experienced during the first fifteen years of life and the display of physical aggression during adulthood. We found that the risk for displaying physical aggression during adulthood was significantly increased by the combination of low MAOA activity and exposure to early trauma.

## Results

### Distribution of allele frequencies and population subgroups

Because MAOA is an X-linked gene, male subjects were straightforwardly assigned to one of two genotype groups: 1) subjects carrying one low activity allele (2-, 3-or 5-repeat form), and 2) subjects carrying one high activity allele (3.5-or 4-repeat form). Females were assigned to three genotype groups: 1) homozygous subjects carrying two low activity alleles, 2) homozygous subjects carrying two high activity alleles, and 3) heterozygous subjects carrying one low and one high activity allele. Expression studies have demonstrated that in female skin fibroblasts the MAOA gene undergoes X-inactivation and shows mono-allelic expression [21]. Although the extent of X-inactivation in human brain is not known, these findings suggest that heterozygous low-high females are mosaic for different MAOA alleles and are likely to have intermediate enzymatic activity. A functional discrimination analysis showed that in our sample the three female genotypes were indistinguishable by physical aggression scores of the Aggression Questionnaire (Wilk's λ = 0.99; F_2,170_ = 0.74, P = 0.47; AQ-PA, see Materials and Methods [22]) and thus, for the purposes of our analysis, we grouped heterozygous female participants together with low-low homozygous females following the convention of a previous study [23]. The low and high enzyme activity groups accounted respectively for 43% and 57% of the total male participants (N = 82) and 54% and 46% of the total female participants (N = 153). Genotype frequencies within the sample did not significantly deviate from those reported for other Caucasian populations (χ^2^ = 2.51, df = 4, P = 0.64) [12], [14].

Participants were divided into two groups according to self-reported exposure to early traumatic life events: 1) those reporting none, and 2) those reporting one or more traumatic events. Thirty-four percent of the participants experienced at least one traumatic life event between 0 and 15 years of age, with some participants reporting the occurrence of multiple traumatic events (Table 1).

**Table 1:** Number of participants and relative percentage who reported early traumatic life events (ETLE) during the first 15 years of their lives*.

Early Traumatic Life Events	
Death of mother	2 (0.84%)
Long absence of the mother due to illness (>100 days)	9 (3.79%)
Absence of the mother due to adoption	1 (0.42%)
Absence of the mother due to upbringing in a foster home	1 (0.42%)
Long absence of the mother due to upbringing by other family kin or unrelated persons	6 (2.53%)
Long absence of the mother due to divorce or separation of parents	0
Death of the father	11 (4.64%)
Long absence of the father due to illness (>100 days)	4 (1.68%)
Absence of the father due to adoption	1 (0.42%)
Absence of the father due to upbringing in a foster home	0
Long absence of the mother due to upbringing by other family kin or unrelated persons	3 (1.26%)
Long absence of the father due to war service or war imprisonment	0
Long absence due to imprisonment	1 (0.42%)
Long absence of the father due to separation or divorce of parents	8 (3.37%)
Separation from parents due to illness of the proband (>100 days)	1 (0.42%)
Severe physical handicap of the subject during childhood	3 (1.26%)
Severe physical handicap of sibling	11 (4.64%)
Parents' marital problems	62 (26.16%)
Alcohol addiction of one or both parents	22 (9.28%)
Severe psychiatric illness of mother or father (other than alcohol dependence)	13 (5.48%)
Violence in the family	32 (13.50%)
Sexual molestation or abuse	8 (3.37%)

* The sum of percentages is greater than 100 because some participants reported more than one ETLE.

Before assessing the effect of ETLE exposure and MAOA genotype on risk for increased physical aggression, we first tested whether subgroups in our sample differed significantly for the distribution of psychiatric diagnosis, ETLE exposure, and MAOA genotype. Such deviations might confound the interpretation of subsequent findings and reveal the existence of gene-by-environment correlation effects. Men and women did not differ in the distribution of psychiatric diagnosis (χ^2^ = 0.92, df = 1, P = 0.34) and exposure to ETLE (χ^2^ = 0.03, df = 1, P = 0.87). Compared to healthy controls, exposure to ETLE between 0 and 15 years of age was more frequent among psychiatric patients (χ^2^ = 3.67, df = 1, P = 0.05). Importantly, the ETLE groups did not differ significantly in MAOA genotype distribution, arguing against the possibility that genotype influenced exposure to traumatic events (χ^2^ = 0.21, df = 1, P = 0.64). Finally, the distribution of MAOA genotype did not differ between the healthy and psychiatric patient groups (χ^2^ = 0.35, df = 1, P = 0.56).

Scores on the AQ-PA scale in our sample ranged from 9 to 41 (median: 14.0). The percentage of psychiatric patients included in the subgroup of participants who scored in the top 25% of the score distribution (AQ-PA ≥ 19) was higher than that included in the subgroup of participants who scored in the bottom 25% (AQ-PA≤11) (50 vs. 28%, χ^2^ = 6.41, df = 1, P<0.01).

### Effects of gender, early traumatic life events, and MAOA genotype on physical aggression

**Figure 1:**
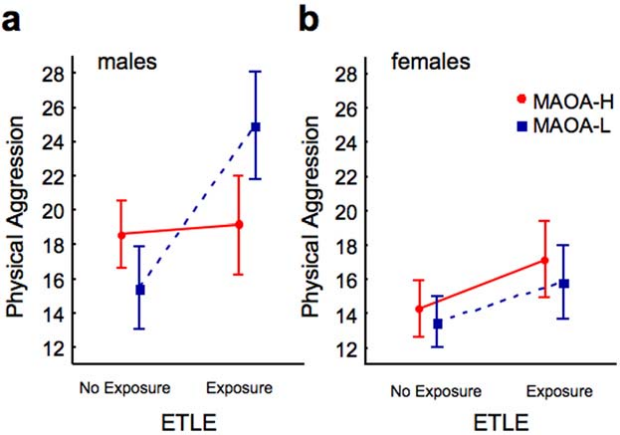
MAOA genotype moderates the association between early traumatic life events and physical aggression. Interactions between gender, MAOA genotype, and early traumatic life events (ETLE) predicted AQ-PA physical aggression scores in (a) males and (b) females. In the male group, carriers of the low, but not the high MAOA activity allele reporting exposure to early traumatic life events showed significantly greater physical aggression scores.

Effects of gender, ETLE, and MAOA genotype were assessed using an ANOVA model with the AQ-PA score as the dependent variable. Statistical analysis revealed significant main effects of gender (F_1,227_ = 36.77, P<0.0001) and ETLE (F_1,227_ = 27.50, P<0.0001), but no main effect of MAOA genotype (F_1,227_ = 0.10, P = 0.75). As expected, physical aggression scores were higher among males and participants reporting exposure to early traumatic life events. The importance of MAOA genotype in modulating the impact of early trauma on adult physical aggression emerged when interaction effects were examined. We found significant MAOA×ETLE (F_1,227_ = 8.20, P = 0.005) and gender×MAOA×ETLE (F_1,227_ = 7.04, P = 0.009) interaction effects. The cumulative variance in the physical aggression score explained by the ANOVA effects involving the MAOA allele was 6.6%. Physical aggression scores were higher in men who had experienced early traumatic life events and who carried low MAOA activity alleles (Table 2 and Figure 1).

**Table 2:** Descriptive statistics and ANOVA results for physical aggression in the entire sample. η_p_^2^: partial eta squared.

Gender	ETLE	MAO-A Activity	Mean	N
Females	No Exposure	High	13.65	43
		Low	13.33	58
		Total	13.47	101
	Exposure	High	16.37	27
		Low	16.40	25
		Total	16.38	52
	Total	High	14.70	70
		Low	14.25	83
		Total	14.46	153
Males	No Exposure	High	19.06	33
		Low	15.09	22
		Total	17.47	55
	Exposure	High	20.36	14
		Low	25.69	13
		Total	22.93	27
	Total	High	19.45	47
		Low	19.03	35
		Total	19.27	82
Total	No Exposure	High	16.00	76
		Low	13.81	80
		Total	14.88	156
	Exposure	High	17.73	41
		Low	19.58	38
		Total	18.62	79
	Total	High	16.61	117
		Low	15.67	118
		Total	16.14	235

Effects: Gender: F_1.227_ = 36.77, p<0.0001, η_p_^2^ = 0.14; ETLE: F_1.227_ = 27.51, p<0.0001, η_p_^2^ = 0.11; MAO-A: F_1.227_ = 0.10, p = 0.75, η_p_^2^ = 0.00; Gender×ETLE: F_1.227_ = 3.28, p = 0.07, η_p_^2^ = 0.01; Gender×MAO-A: F_1.227_ = 0.24, p = 0.62, η_p_^2^ = 0.00; ETLE×MAO-A: F_1.227_ = 8.20, p = 0.005, η_p_^2^ = 0.04, Gender×ETLE×MAO-A: F_1.227_ = 7.04, p = 0.009, η_p_^2^ = 0.03.

We repeated the ANOVA in the subgroup of healthy volunteers (N = 145) to exclude that the G×E interactions we found were due to the inclusion of psychiatric patients in our sample and were not generalizable to the population at large. The results for the subgroup of healthy volunteers were identical to those for the entire sample. We found significant main effects of gender (F_1,137_ = 37.53, P<0.0001) and ETLE (F_1,137_ = 10.34, P = 0.002), no main effect of MAOA genotype (F_1,137_ = 0.86, P = 0.35), and significant interaction effects for MAOA×ETLE (F_1,137_ = 10.13, P = 0.002) and for gender×MAOA×ETLE (F_1,137_ = 6.66, P = 0.01). The cumulative variance in the physical aggression score explained by the ANOVA effects involving the MAOA allele was 12.1% (Table 3).

**Table 3:** Descriptive statistics and ANOVA results for physical aggression in the sub-sample of healthy volunteers. η_p_^2^: partial eta squared.

Gender	ETLE	MAO-A Activity	Mean	N
Females	No Exposure	High	12.46	26
		Low	12.69	36
		Total	12.60	62
	Exposure	High	13.86	14
		Low	15.13	15
		Total	14.52	29
	Total	High	12.95	40
		Low	13.41	51
		Total	13.21	91
Males	No Exposure	High	19.09	22
		Low	14.95	19
		Total	17.17	41
	Exposure	High	17.75	8
		Low	23.60	5
		Total	20.00	13
	Total	High	18.73	30
		Low	16.75	24
		Total	17.85	54
Total	No Exposure	High	15.50	48
		Low	13.47	55
		Total	14.42	103
	Exposure	High	15.27	22
		Low	17.25	20
		Total	16.21	42
	Total	High	15.43	70
		Low	14.48	75
		Total	14.94	145

Effects: Gender: F_1.137_ = 37.53, p<0.0001, η_p_^2^ = 0.22; ETLE: F_1.137_ = 10.33, p = 0.002, η_p_^2^ = 0.07; MAO-A: F_1.137_ = 0.86, p = 0.36, η_p_^2^ = 0.01; Gender×ETLE: F_1.137_ = 1.01, p = 0.32, η_p_^2^ = 0.01; Gender×MAO-A: F_1.137_ = 0.00, p = 0.96, η_p_^2^ = 0.00; ETLE×MAO-A: F_1.137_ = 10.13, p = 0.002, η_p_^2^ = 0.07, Gender×ETLE×MAO-A: F_1.137_ = 6.66, p = 0.01, η_p_^2^ = 0.05.

## Discussion

We studied a mixed population of psychiatric outpatients and healthy volunteers in order to examine the gene-environment interaction effect of MAOA genotype and early trauma on the increased risk for self-reported levels of physical aggression during adulthood. We found that levels of physical aggression were significantly higher in men who had experienced traumatic events during the first 15 years of life and who carried the low expression allele (MAOA-L). When we repeated the analysis in the sub-sample of healthy volunteers, the results did not change. Our results are consistent with the majority of previous reports [14]–[17], [24] and point toward the MAOA genotype as a genetic factor that moderates the impact of early traumatic life events on the developmental pathway leading to later-life aggression.

Based on the findings of several independent studies, the relationship between the MAOA polymorphism and aggression appears to be fairly consistent. Yet, the intervening neural and psychological mechanisms are still unclear. One promising line of research has investigated the possibility that individuals with the low expression allele might be more sensitive to negative social experiences, which might ultimately result in defensively aggressive behavior. A recent fMRI study showed that the MAOA genotype at risk for impulsivity and violent behavior is associated with reduced gray matter volumes in limbic regions such as the amygdala, dorsal anterior cingulated cortex (dACC), and subgenual ACC and greater amygdala and subgenual ACC responsivity to negative emotional faces [25]. Consistent with the social hypersensitivity hypothesis, Eisenberger et al. (2007) have found that, compared to individuals with the high expression allele, healthy men and women with the MAOA-L reported higher trait aggression, higher interpersonal sensitivity and showed greater dorsal anterior cingulated cortex activity (associated with rejection-related distress) in response to social exclusion [26]. If replicated by future studies, these preliminary findings suggest that the MAOA-L would confer a vulnerability to negative social experiences, including early trauma, and a specific proclivity toward reactive aggression, i.e. that type of aggression triggered by exaggerated levels of negative emotion, such as anger and anxiety.

Although recent research has demonstrated that the allele×environment strategy is promising for detecting individual vulnerability to environmental risk, reported associations between gene variants and complex behaviors are in general weak. This is likely to reflect the fact that a number of several alleles contribute in various small ways to the interplay between environmental risk factors and development of complex behaviors. In the case of G×E interactions involving MAOA alleles and predicting antisocial behavior and/or conduct disorder, effect sizes reported to date are small on average. In this regard, the present study is no exception. The cumulative variance in the physical aggression score explained by the ANOVA effects involving the MAOA allele was 6.6% in the entire sample and 12.1% in the sub-sample of healthy volunteers. However, the significant G×E interaction effects found in the present study are remarkable considering its relatively small sample (N = 235) and require a methodological comment.

The questionnaire we used to measure the occurrence of early traumatic life events explored a variety of adverse experiences other than physical and sexual abuse. There is a large body of evidence in the clinical literature demonstrating that childhood attachment-related trauma [27], [28], such as prolonged separation from parents or chronic conflict within the family (which were the most frequently reported events among the participants of our study), and lack of parental warmth [29] can increase the risk for aggressive behavior during adolescence and adulthood. The exclusive or limited focus on physical abuse and maltreatment might in part explain the failure of some previous studies to confirm the role of MAOA genotype in moderating the relationship between early stress and subsequent aggressive behavior [18]–[20].

Another important difference between the present study and previous ones is the measure used to assess the behavioral phenotype. As dependent variable, we used the physical aggression scale of the Aggression Questionnaire [22], a continuous measure that assesses individual propensity toward aggression and that can be administered to individuals in the normative range of aggressive behavior. In contrast, most previous studies have used relatively indirect measures of aggressive behavior, such as incarceration, criminal conviction, or a diagnosis of antisocial behavior or conduct disorder. The problem with these measures is that antisocial behavior or conduct disorder include a number of behaviors other than aggression and that they are dichotomous (presence or absence of the behavioral phenotype), which may lead to a corresponding loss of discriminatory power. Our findings suggest that the use of psychometric tools assessing behavioral aspects of aggression ranging from normative levels to pathological extremes may increase the likelihood of detecting interactions of MAOA genotype with early traumatic events.

This study has several limitations, the first of which is the small sample size. Studies relating genetic polymorphisms to behavioral or self-report assessments typically use much larger samples. Thus, the present results should be interpreted with caution until these findings have been replicated in larger samples. However, the within-study replication in psychiatric patients and healthy volunteers justifies a fairly high level of confidence. Second, we used a retrospective measure to assess the occurrence of traumatic events during childhood. When the type of events assessed is not limited to objective trauma (e.g., death of mother) but also includes perceived situations (e.g., parental marital problems), retrospective data may be subject to faulty recall or systematic distortions, even though the construct validity of our measure of early trauma was supported by its strong association with later-life aggression, in accordance with most other studies related to this topic. Although prospective studies are clearly desirable in delineating the role of early environment in the development of later-life aggressive behavior, such investigations face daunting methodological difficulties. Until such methodological problems can be overcome, reliance will have to be placed on a variety of sources of information, including retrospective data, to study G×E. Third, we used a self-report measure of aggression, and the social desirability bias of some subjects might have affected their self-reporting of aggression. Subjects motivated by need for social approval may not report as much aggression as those to whom social approval is less important. Such a bias, however, probably reduced our ability to detect interaction effects and thus implies that the findings are conservative estimates of G×E. Fourth, we included women in our sample to assess the impact of gender on the MAOA-early trauma interaction. Few studies investigating MAOA-related aggression have included female subjects [25], [26]. Inclusion of women in such studies is complicated by the fact that somatic MAOA allele mosaicism, due to X-chromosome inactivation, makes classification of heterozygous females problematic. Previous research has shown that female heterozygotes show patterns of neural activity intermediate between MAOA-L and MAOA-H male hemizygotes and that female homozygotes show patterns of neural activity comparable to male hemizygotes [25]. Because, in our sample physical aggression scores were indistinguishable among the three female genotypes, we were not able to draw conclusions about the status of heterozygous female subjects.

In conclusion, despite its limitations, this study supports and extends the findings of previous reports showing that the MAOA polymorphism in combination with early experience modulates individual proclivity to later-life physical aggression. Its major methodological contribution is that the use of continuous self-report measures of aggression ranging from normative levels to pathological extremes and a wider focus in assessing early trauma may increase the likelihood of detecting interactions between the MAOA gene and childhood environment.

## Materials and Methods

### Population sample and recruitment

The population sample of this study consisted of 90 outpatients (69% women; mean±SD age: 32.18±8.66 years) consecutively admitted to the day hospital of the psychiatric clinic at the University of Rome Tor Vergata and 145 healthy volunteers (63% women; mean±SD age: 29.59±6.73 years). All participants were of Caucasian origin. Diagnostic assessment was made by experienced clinical psychiatrists using the Structured Clinical Interview for DSM-IV Axis I Disorders (SCID-CV) [30] and the Schedule for Interviewing DSM-IV Personality Disorders-IV (SIDP-IV) [31]. Patients with medical or neurological disorders, mental retardation, or psychotic disorders were excluded from the sample. The diagnostic composition of the clinical group was as follows: anxiety disorders, 28%; major depressive disorder, 27%; eating disorders, 21%; cluster B personality disorders, 13%; bipolar disorder, 11%. All patients were interviewed when they had partially improved and reached the drug treatment dosage prescribed for maintenance therapy. Healthy volunteers were recruited among students in the medical school, paramedic staff members, and conscripts of the Italian army. Inclusion in the control group required the absence of current or past psychiatric disorders, as confirmed by diagnostic interview.

All data were obtained under informed consent and using procedures and protocols approved by the University of Rome Tor Vergata Intramural Ethics Committee and the EMBL Bioethics Internal Advisory Committee (BIAC).

### DNA extraction and genotyping

For all participants DNA was obtained from buccal samples [32] and DNA was extracted using standard procedures. PCR was carried out using the following conditions: initial 5-min denaturing step at 95.0 C, followed by 35 cycles at 94.0 C for 1 min, 53.8 C for 1 min and 72.0 C for 1 min 30 s, and a final extension phase at 72.0 C for 10 min. Primer sequences were those described by Sabol et al. (1998): MAO-F (5′-ACAGCCTGACCGTGGAGAAG-3′) and MAO-R (5′-GAACGGACGCTCCATTCGGA-3′). Reactions were performed in 25 µl total volume with 50 ng genomic DNA, 1.5 mM MgCl_2_, 10 pmoles of each primer, 0.33 mM dNTPs, and 1.5 U of native Taq (Promega, Madison, WI). PCR products were assayed on a 3% agarose gel. The primers used yielded 290, 320, 335, 350 and 380 bp fragments corresponding to the 2-, 3-, 3.5-, 4-and 5-repeat alleles, respectively [12].

### Assessment of physical aggression

On the same day when DNA was collected, each subject completed the Aggression Questionnaire (AQ) [22]. AQ is a 29-item scale divided into four subscales: physical aggression (9 items), verbal aggression (5 items), anger (7 items), and hostility (8 items). Each statement was assessed by a five-point Likert scale (from 1 = never or hardly ever applies to me, to 5 = very often applies to me). Two items were reverse scored. In addition to the scores on each subscale, a total score was also calculated. AQ measures aggressive attitudes that are consistent across an extended time frame and characterological in nature, and is therefore considered a trait measure of aggression [33]. AQ is related to other self-report measures of aggression as well as behavioral indicators of aggression. The AQ subscales have high levels of internal consistency and moderate to high levels of test-retest reliability [34]. In the present study, data analysis was based on the physical aggression (AQ-PA) subscale. Statements rating AQ-PA included: “I have become so mad that I have broken things”, “Once in a while I can't control the urge to strike another person”, “Given enough provocation, I may hit another person” and “If somebody hits me, I hit back”.

### Assessment of early traumatic life events

The occurrence of traumatic life events during childhood was assessed using a questionnaire developed by Bandelow et al. [35] to measure exposure to a variety of adverse early experiences including: separation from father/mother due to death or long absence (>100 days) associated with illness, adoption, or separation or divorce of parents; occurrence of severe handicap in the subject or subject's siblings; severe parental marital problems; parental mental illness, alcoholism, or violence in the family (including physical abuse of the subject); and sexual abuse of the subject. Prevalence of early traumatic life events (ETLE) occurring between 0 and 15 years of age was recorded (Table 1).

### Statistical analysis

Functional discrimination analysis was used to detect differences in levels of physical aggression among genotype groups of female participants. Chi-square tests were used to test differences between categorical variables. Main and interaction effects of gender, ETLE, and MAOA genotype on the physical aggression score were calculated by using three-way analysis of variance (ANOVA). All statistical analyses were carried out with the help of Statistica (StatSoft, Tulsa, OK) and SPSS (SPSS, Chicago, IL) software.
